# Toward Better Risk Stratification for Implantable Cardioverter-Defibrillator Recipients: Implications of Explainable Machine Learning Models

**DOI:** 10.3390/jcdd9090310

**Published:** 2022-09-17

**Authors:** Yu Deng, Sijing Cheng, Hao Huang, Xi Liu, Yu Yu, Min Gu, Chi Cai, Xuhua Chen, Hongxia Niu, Wei Hua

**Affiliations:** The Cardiac Arrhythmia Center, State Key Laboratory of Cardiovascular Disease, Fuwai Hospital, National Center for Cardiovascular Diseases, Chinese Academy of Medical Sciences & Peking Union Medical College, No.167 North Lishi Road, Beijing 100037, China

**Keywords:** implantable cardioverter-defibrillators, machine learning, mortality, first appropriate shock, Shapley Additive exPlanations values, personalized risk stratification

## Abstract

**Background:** Current guideline-based implantable cardioverter-defibrillator (ICD) implants fail to meet the demands for precision medicine. Machine learning (ML) designed for survival analysis might facilitate personalized risk stratification. We aimed to develop explainable ML models predicting mortality and the first appropriate shock and compare these to standard Cox proportional hazards (CPH) regression in ICD recipients. **Methods and Results:** Forty-five routine clinical variables were collected. Four fine-tuned ML approaches (elastic net Cox regression, random survival forests, survival support vector machine, and XGBoost) were applied and compared with the CPH model on the test set using Harrell’s C-index. Of 887 adult patients enrolled, 199 patients died (5.0 per 100 person-years) and 265 first appropriate shocks occurred (12.4 per 100 person-years) during the follow-up. Patients were randomly split into training (75%) and test (25%) sets. Among ML models predicting death, XGBoost achieved the highest accuracy and outperformed the CPH model (C-index: 0.794 vs. 0.760, *p* < 0.001). For appropriate shock, survival support vector machine showed the highest accuracy, although not statistically different from the CPH model (0.621 vs. 0.611, *p* = 0.243). The feature contribution of ML models assessed by SHAP values at individual and overall levels was in accordance with established knowledge. Accordingly, a bi-dimensional risk matrix integrating death and shock risk was built. This risk stratification framework further classified patients with different likelihoods of benefiting from ICD implant. **Conclusions:** Explainable ML models offer a promising tool to identify different risk scenarios in ICD-eligible patients and aid clinical decision making. Further evaluation is needed.

## 1. Introduction

Since their first introduction in clinical practice four decades ago, implantable cardiac defibrillators (ICDs) have been undoubtedly recognized as an effective modality to prevent sudden cardiac death (SCD), which affects millions of people worldwide each year [1,2,3]. Nonetheless, as heterogeneity exists among patients, and a high proportion of ICD recipients in the real-world did not receive appropriate device therapies in the long-term follow-up [4,5,6]. Moreover, patients implanted with ICD may suffer from procedure-related complications, inappropriate ICD shocks, and psychological disorders [7]. As such, to classify those most likely to benefit from ICD therapy, personalized risk assessment is warranted.

Efforts have never ceased to optimize risk stratification for ICD candidates. Previous established models, such as the Seattle Heart Failure Model–D [8], the Seattle Proportional Risk model [9,10], and other models [11,12,13,14,15,16,17] integrating both risks of ventricular arrhythmias and pump failure death have been proved to have acceptable discrimination to identify potential beneficiaries. These tools built by traditional statistical modeling strategies are easy to interpret and use. However, statistical modeling is not suitable for handling complex interactions and high-order nonlinear relationships that exist in real situations [18]. In contrast, machine learning (ML), inherently embedded to deal with these problems, may be a feasible solution to improve risk prediction [18]. Along with its high flexibility and accuracy, machine learning has also been questioned regarding its low interpretability, which is of particular importance in healthcare settings. Nowadays, with advances in explainable ML tools, such as the Shapley Additive exPlanation (SHAP) values, it is possible to have a thorough understanding of ML models [19].

In this study, we compared machine learning approaches incorporating time-to-event analysis to traditional Cox proportional hazards (CPH) modeling for all-cause death and appropriate shock prediction. We also used SHAP values to explain each variable’s contribution to outcome events. Finally, we suggested a feasible framework for risk stratification by integrating those two dimensions of risk of death and shock.

## 2. Materials and Methods

### 2.1. Patient Population

**Figure 1:**
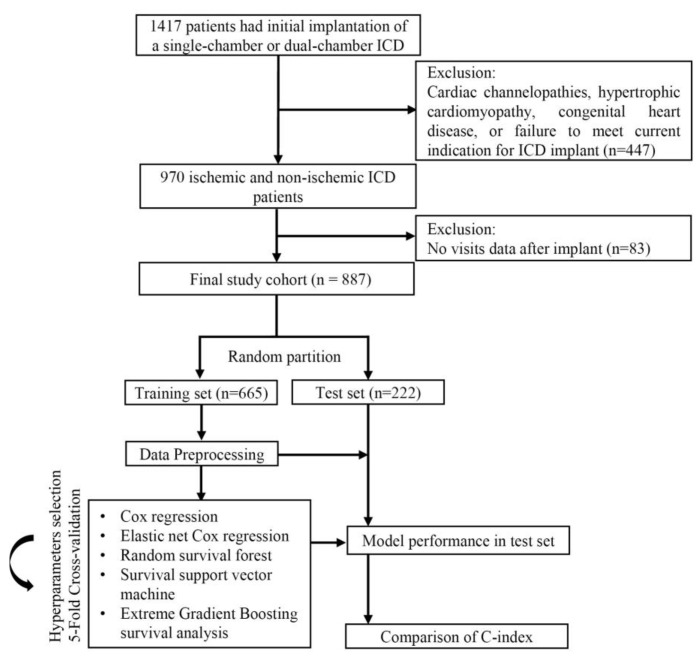
Study flow diagram. ICD, implantable cardioverter defibrillation.

We retrospectively enrolled 1417 adult patients who received initial single- or dual- chamber ICD implantation from 1 January 2010, through 31 December 2020, at our institution. We excluded patients with specialized indications for ICDs (n = 447), including cardiac channelopathies, hypertrophic cardiomyopathy, and congenital heart disease due to their distinctive pathophysiology, and patients who did not meet the current indication for ICD implant [3]. We also excluded patients without any visit data after implant (n = 83). After the exclusion of these patients, 887 patients were retained for analysis. This study complied with the Declaration of Helsinki and was approved by Ethics Committee of Fuwai Hospital. All patients provided informed consent. The flowchart of the study, including patient selection, is illustrated in Figure 1.

### 2.2. Outcome Definition

All-cause death and the first appropriate ICD shock were both predefined endpoints. Survival conditions were obtained by hospital records, death certification, or phone calls to patients’ relatives. Patients were required to perform device interrogations every 6–12 months and shortly after perceiving device therapies. Appropriate ICD shock was defined as ICD shock delivered for ventricular tachycardia or ventricular fibrillation and was adjudicated by a trained electrophysiologist though the therapy zones were at the discretion of the treating physicians. The censoring dates for the two endpoints were not necessarily the same because survival status was further ascertained even after the last follow-up of device interrogations.

### 2.3. Data Collection and Preprocessing

A total of 45 variables, including patient demographics, laboratory values, comorbidities, medications, electrocardiogram findings, and echocardiographic indices were collected from electronic medical records. Fourteen variables with missing data are shown in Table S1. All these variables had missing rates less than 5%. Data preprocessing was performed using the scikit-learn (version 1.0.2) module in Python 3.7.13. Dichotomous variables were encoded using the OneHotEncoder function. The New York Heart Association (NYHA) functional classification was transformed to an ordinal variable using the OrdinalEncoder function. Continuous variables were scaled to normal distribution through Box-Cox transformation and standardization using the PowerTransformer function, to diminish the impact of variable variances on model performance. Missing values were imputed with the mean value of each variable by the SimpleImputer function. Raw data and the corresponding transformed values are provided in Table S2.

### 2.4. Modeling Strategies

The modeling process of all-cause death and appropriate shock was independent. All patients enrolled were randomly split into a training set (n = 665, 75%) and a test set (n = 222, 25%) using a stratified sampling method based on outcome events. To avoid data leakage, data preprocessing was performed after data partitioning. We modeled the outcomes as right-censored data. Four ML algorithms, including elastic net Cox regression (EN-Cox), random survival forests (RSF), survival support vector machine (SSVM), and eXtreme Gradient Boosting (XGBoost), were applied to build prediction models using the scikit-survival [20] (version 0.17.1) and XGBoost [21] (version 1.5.2) modules. To ensure the best performance of each model, hyperparameter tuning techniques of grid-search with five-fold cross-validation were used to obtain optimal hyperparameters. On the other hand, the CPH model was fitted through stepwise backward Cox regression based on Akaike’s information criterion by entering variables significantly related to outcomes at a *p* < 0.10 level using the ‘rms’ package of R software 4.1.2. It was set as a benchmark for model comparison. The best ML models were respectively selected to construct risk scores predicting death and shock. Subsequently, patients were respectively divided into three equal-sized risk groups (low, intermediate, high) of death and shock. Accordingly, a 3-by-3 risk assessment matrix accounting for both risks was built. This risk stratification system ultimately identified patients with different likelihood of benefiting from ICD implant.

### 2.5. Model Interpretability

SHAP values, based on the coalitional game theory by Lundberg and Lee [19], were used to explain each variable’s contribution to prediction (risk scores in this study). It provides both global interpretability (to which extent each predictor contributes positively or negatively to the outcome event on the average) and local interpretability (contribution of each predictor to the outcome in an individual). SHAP summary plots and dependence plots were used to illustrate global interpretability, whereas the force plot was used to illustrate local interpretability.

### 2.6. Statistical Analysis

Continuous variables were summarized as either the mean ± standard deviation or the median with the interquartile range (IQR), as appropriate; categorical variables were presented as frequencies and percentages. Baseline characteristics were compared using the Student’s *t*, the Mann–Whitney U, the chi-square or the Fisher exact tests, as appropriate. Survival curves were plotted using the Kaplan–Meier estimator. Log-rank tests were used to compare unadjusted differences between groups. Model accuracy was assessed on the test set using Harrell’s concordance index (C-index) owing to its independence of the proportional-hazards assumption. The C-index ranges from 0 to 1, with 1 representing perfect discrimination between subjects who experience the outcome or not, with 0 representing a completely wrong model. The confidence intervals of the C-index were estimated by the bootstrap method with 100 resamplings. The overall difference of the C-index was tested using one-way ANOVA. Furthermore, the C-index of each ML model was compared with the CPH model using the Dunnett’s test. In sensitivity analysis, we imputed missing variables by multivariable imputation using Bayesian ridge regression in an iterative method. A two-sided *p*-value ≤ 0.05 was considered statistically significant.

## 3. Results

### 3.1. Patient Characteristics

A total of 887 patients who had initial ICD implant were identified in the study. The mean age at ICD implantation was 59.0 ± 13.0 years. Patients were predominantly male (75.2%), with a secondary prevention indication (72.9%), and had no pacing indications (93.3%). Approximately half had ischemic cardiomyopathy (48.8%). Most patients prescribed β-blockers and renin–angiotensin–aldosterone system (RAAS) inhibitors, while only 10.6% were prescribed calcium channel blockers. Patient characteristics before and after the partition are summarized in Table 1, with all covariates included in the prediction models. Compared to the test set, there were no significant differences except for higher dual-chamber ICD use and blood urea nitrogen (BUN) levels in the training set for the prediction of death. On the other hand, patients in both sets for the prediction of appropriate shock had similar characteristics across all spectra.

### 3.2. Outcome Events

During the study period, 199 patients died, with a median follow-up duration of 4.8 (IQR, 3.0–7.1) years (incidence rate of 5.0 per 100 person-years); 265 patients received the first appropriate shock with a median follow-up of 2.7 (IQR, 1.1–5.1) years (12.4 per 100 person-years).

### 3.3. Prediction of All-Cause Death and Appropriate Shock

**Figure 2:**
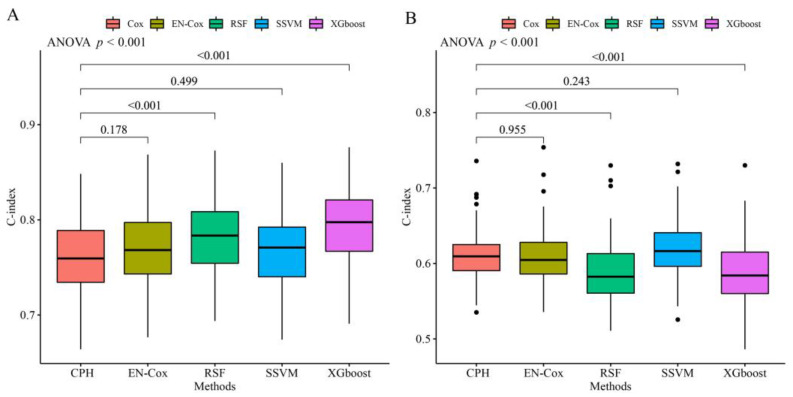
Comparison of C-index between CPH and ML algorithms for all-cause death (**A**) and first appropriate shock (**B**) in the test set. For predicting all-cause death, the C-index of CPH, EN-Cox, RSF, SSVM, and XGBoost were 0.760 (95% CI 0.752–0.768), 0.771 (95% CI 0.763–0.779), 0.781 (95% CI 0.773–0.788), 0.767 (95% CI 0.759–0.775), and 0.794 (95% CI 0.786–0.802), respectively. For predicting shock, the C-index of CPH, EN-Cox, RSF, SSVM, and XGBoost were 0.611 (95% CI 0.604–0.618), 0.608 (95% CI 0.601–0.615), 0.589 (95% CI 0.581–0.597), 0.621 (95% CI 0.613–0.628), and 0.588 (95% CI 0.580–0.596), respectively. CI, confidence interval; CPH, Cox proportional hazards regression; other abbreviations as in Table 2.

ML and CPH models were developed in the training set. Table 2 shows the parameter search space and optimal parameters for the ML models. Model performance in the test set is shown in Figure 2. For the prediction of death, the CPH model achieved a C-index of 0.760 (95% CI 0.752–0.768) on the test set. Among ML algorithms, XGBoost and RSF both showed a significantly greater C-index than the CPH model (C-index difference of 0.034 and 0.021, respectively, both *p* < 0.001). The EN-Cox model had a trend toward better performance than the CPH model (C-index difference of 0.011, *p* = 0.178). For the prediction of appropriate shock, SSVM had the highest C-index of 0.621 (95% CI 0.613–0.628), but it was not significantly higher than the CPH model (C-index difference of 0.009, *p* = 0.243). EN-Cox had similar discrimination compared to the CPH model. However, XGBoost and RSF were not superior to the CPH model (C-index difference of −0.023 and −0.022, respectively, both *p* < 0.001). Model performance in the training set is provided in Table S3.

### 3.4. Explainability Based on SHAP Values

**Figure 3:**
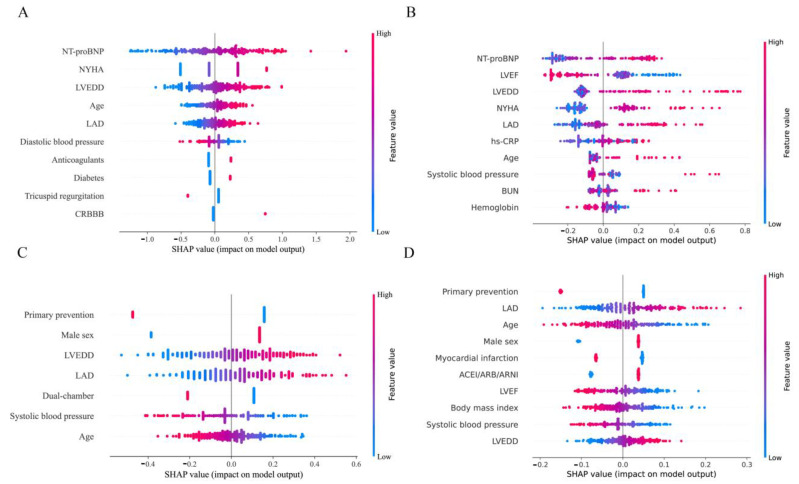
Model interpretability using SHAP values. CPH model (**A**), XGBoost (**B**) for prediction of death; CPH model (**C**), SSVM (**D**) for prediction of appropriate shock. The X-axis stands for SHAP value, and the predictor lies orderly on the Y-axis according to their importance (the higher in position, the more important). Only the top 10 important predictors are left in the plot. Each point on the summary plot represents a single predictor of an individual. Overlapping points are jittered in the y-axis. Red and blue colors respectively indicate higher and lower values of a predictor. For example, a high value of NT-proBNP increases the risk score of death in the CPH model predicting death. In other words, it is a risk factor. Conversely, a high value of diastolic blood pressure reduces the death risk score, making it a protective factor. SHAP, SHapley Additive exPlanations; other abbreviations as in Figure 2 and Table 1.

**Figure 4:**
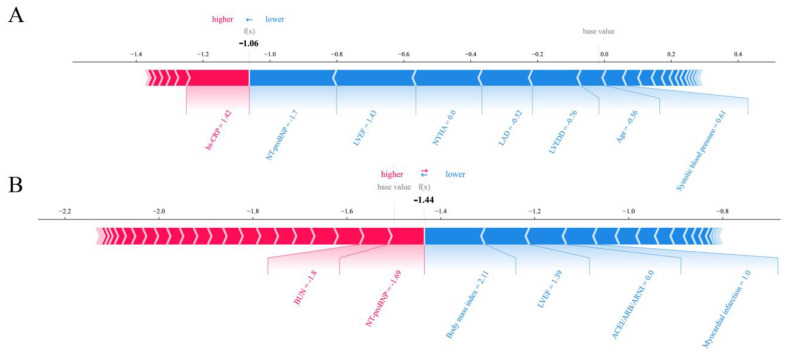
SHAP force plots for XGBoost predicting all-cause death (**A**) and SSVM predicting shock (**B**) in a single patient. Red and blue bars respectively represent the positive and negative effects of each predictor contributing to the occurrence of the outcome. The extent of impact is represented by the size of the bar. Bold values represent the predicted risk scores. Of note, values have been transformed and raw values can be seen in Table S2. Abbreviations as in Table 1, Table 2, and Figure 3.

We computed the SHAP values of CPH and ML models in the test set. As illustrated in Figure 3 and Figure S1, for the XGBoost model predicting death, increased N-terminal pro-brain natriuretic peptide (NT-proBNP), left ventricular end-diastolic diameter (LVEDD), the New York Heart Association (NYHA) functional classification, left atrial diameter (LAD), high-sensitivity C-reactive protein (hs-CRP), age, BUN, and abnormal systolic blood pressure (< 110 mm Hg or > 140 mm Hg) were the most important risk factors. In contrast, increased left ventricular ejection fraction (LVEF) and hemoglobin level were among the most protective factors. For SSVM predicting shock risk, the primary prevention indication, increasing age, previous myocardial infarction, and higher LVEF, body mass index, and systolic blood pressure were the most protective factors, while male sex, usage of RAAS inhibitors, increased LAD, and LVEDD were the greatest risk factors (Figure 3 and Figure S2). SHAP summary plots show that each predictor in CPH models had the same effect direction as the regression coefficients (Table S4). The summary plots of other ML algorithms are shown in Figure S3. In addition, Figure 4 illustrates how each variable contributes to the outcome prediction in a single patient.

### 3.5. Establishment of Bi-Dimensional Risk Profiles

**Figure 5:**
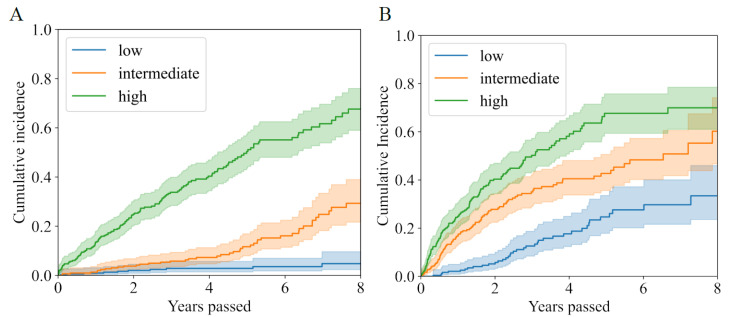
Cumulative incidence curves of all-cause death (**A**) and first appropriate shock (**B**) by risk stratum.

**Figure 6:**
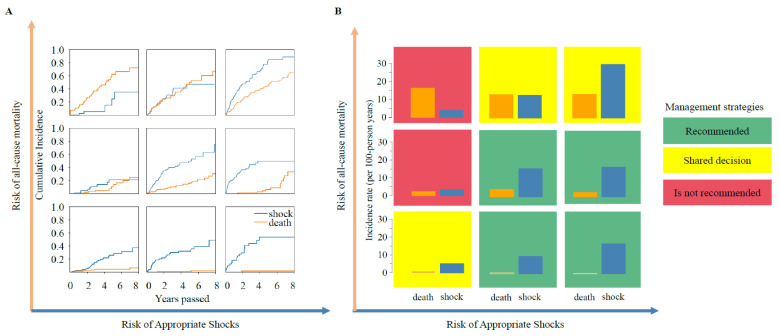
The construction of bi-dimensional risk profiles. (**A**), The cumulative incidence curves. (**B**), Bar plot of the annual incidence rate. The X-axis and Y-axis represent the three increasing risk strata of appropriate shock and all-cause death, respectively. A 3*3 risk profile was built accordingly. As a result, three different treatment strategies might be considered.

XGBoost and SSVM were respectively chosen to construct the risk model for all-cause death and appropriate shock. Patients were classified into three increasing risk categories of all-cause death and appropriate shock by XGBoost and SSVM (Figure 5), respectively. Accordingly, 3*3 risk profiles were developed (Figure 6). Patients with the highest risk of death (16.58 per 100 person-years) and lowest risk of shock (3.99 per 100 person-years) may not benefit from ICD implant. Conversely, patients with the lowest risk of death (0.39 per 100 person-years) and highest risk of shock (16.99 per 100 person-years) may benefit from implant. For those patients with both high risk of death and shock, low risk of death and marginally high risk of shock, shared decisions between patients and clinicians are needed. Strategies need to be made in accordance with risk scenarios.

### 3.6. Sensitivity Analysis

Instead of mean imputation, Bayesian ridge regression was used to impute missing values. Data partitioning, preprocessing, and hyperparameter search spaces were kept the same. The results supported the primary analysis. The best hyperparameters (Table S5) selected did not substantially deviate from the primary analysis. Model performance was compared and is shown in Figure S4. For the prediction of death, XGBoost also showed better performance than the CPH model (C-index difference of 0.025, *p* < 0.001). For the prediction of shock, SSVM also had better performance than the CPH model, although not statistically different (C-index difference of 0.011, *p* = 0.103). The explanation of the models predicting death and shock is shown in Figure S5.

## 4. Discussion

We leveraged a single-center ICD cohort to create CPH and ML models that predict all-cause death and appropriate shock with an average of nearly 5-year follow-up. We demonstrated that optimized ML models had comparable or better performance than CPH models. SHAP plots further showed that traditional risk factors in ML models had explainable predictive value consistent with established knowledge. Ultimately, we raised a feasible framework to classify ICD patients into a 3*3 matrix of risk scenarios, which may facilitate individualized risk stratification. To the best of our knowledge, this is the first head-to-head study comparing survival ML algorithms with the statistical CPH in ICD patients.

Risk scores using statistical modeling strategies have shown satisfactory performance in ICD benefit prediction [8,9,10,11,12,13,14,15,16,17,22,23]. Survival analysis using CPH regression is a standard paradigm for identifying risk factors and predicting prognosis. However, it only works under two key assumptions: the proportional hazard assumption and the linearity assumption. In comparison, ML approaches do not rely on prior hypotheses and assumptions and therefore are highly flexible [18]. In addition, ML algorithms can handle complex interactions in large datasets in which CPH regression may fail to converge [24]. To date, ML algorithms have been widely applied to cardiovascular disease, from arrhythmias identification to electro-anatomical mapping and to clinical decision support and prognosis prediction [18]. In this study, ML-based models also had an excellent performance in the outcome prediction of ICD recipients.

We demonstrated that XGBoost had the highest discrimination among ML methods in all-cause death prediction and outperformed the CPH model. We further utilized SHAP values to interpret the model. As it showed, older age, higher NT-proBNP, LVEDD, LAD, hs-CRP, BUN, and worsening heart functional status assessed by NYHA were among the most important risk factors. Conversely, higher LVEF and hemoglobin were the most protective factors. These results were in line with previous findings, as deteriorated heart and renal function were related to an increased risk of death [11,16,17,25,26,27]. Additionally, hs-CRP, a biomarker of acute inflammation, was an established risk factor [28]. Systolic blood pressure was also one of the most predictive factors, with a U-shaped relationship shown in the SHAP dependence plot. Death risk increased at both low and high systolic blood pressure, highlighting the role of blood pressure control on mortality. This phenomenon was overlooked in previous studies [11,25].

On the other hand, predicting appropriate ICD shock was much harder. Although we demonstrated SSVM achieved the highest accuracy, it was only minimal to moderate. Expectedly, prevention indication ranked first among various clinical characteristics, underlining its importance to SCD risk [3]. Consistent with previous studies, male sex, higher LAD, and LVEDD were related to an increased risk of shock [9,11,15,22], while myocardial infarction, increasing age, LVEF, body mass index, and systolic blood pressure were related to a decreased risk [9,11,17,29]. However, compared to the general consensus [30], the use of RAAS inhibitors was associated with an increased shock risk. This might partly be attributed to the data-driven nature of ML algorithms and does not necessarily represent a causal relationship. Of note, this effect was not found in other ML algorithms predicting shock risk (Figure S3D–F). As a result, more caution is needed when choosing the right model in the clinical setting.

Our results outlined the difficulties and complexities of predicting ICD shock, which was a surrogate for life-threatening ventricular arrhythmias. It has been widely accepted that the development of ventricular tachycardia/ventricular fibrillation is a dynamic and evolving process involving the participation of multiple pathophysiological processes [22,31,32]. Abnormal heart function, electrical instability, genetic mutations, autonomic dysregulation, and comorbidities have been found to contribute to an increased risk of SCD [7,33,34]. Nonetheless, evidence was inconclusive because of conflicting results, limiting their utilization in clinical settings [7,33,34]. Specifically, several factors may contribute to the low accuracy of this study. First, our cohorts mainly comprised patients with secondary prevention. Previous studies have demonstrated the failure to identify risk factors and establish a risk model in these patients [35,36]. Second, a paucity of cardiac magnetic resonance imaging (MRI) data also impaired the performance. Myocardial replacement fibrosis detected by late gadolinium enhancement is more strongly associated with SCD than LVEF in both ischemic and nonischemic cardiomyopathy [23,29,32,37]. Furthermore, a broader scar zone size is related to a greater SCD risk [27,29,37]. T1-mapping techniques might also add information to arrhythmogenesis [33,34]. Third, ICD programming was left at the discretion of the operators. A standard protocol was not applied. In conclusion, ML is not a panacea. On the contrary, model performance largely depends on prior knowledge and data quality.

Disregarding the mode of death (pump failure or sudden death) may lead to incomplete risk assessment and subsequent biased clinical decisions to an ICD candidacy. Therefore, we developed a framework by integrating the risk of all-cause death and shock and finally identified nine risk profiles. Patients with the highest risk of death and the lowest risk of shock may not benefit from ICD implant. As a result, an ICD may be deferred in this scenario. On the other hand, for those with the lowest risk of death but the highest risk of shock, an ICD implant is justified. For patients with both the highest risk of shock and death, shared decisions between healthcare providers and patients are encouraged, as ICD is solely amenable to shockable arrhythmic events instead of non-shockable rhythms or pump failure death. Additionally, a comprehensive evaluation must be implemented before decision-making in other scenarios.

In fact, this risk assessment framework was first introduced by Buxton et al. [38]. A total of 25 baseline variables including the electrophysiological study result were collected in 674 patients with coronary artery disease enrolled in the MUSTT (Multicenter Unsustained Tachycardia Trial) study [38]. Risk scores of all-cause death and arrhythmic death were built, with satisfactory C-indexes of 0.78 and 0.70, respectively. Later, Lee et al. [11] also developed similar dual risk stratification models using 3445 primary prevention ICD patients by competing risk analysis. More recently, Reeder et al. [17] and Younis et al. [15] also built such models using data from the SCD-HeFT (Sudden Cardiac Death in Heart Failure Trial) and MADIT (Multicenter Automatic Defibrillator Implantation) trial. The former had a C-index for the non-arrhythmic mortality of 0.68 and ventricular tachycardia/ventricular fibrillation of 0.71 [15]. The latter had an area under the curve at 5 years for death of 0.79 and ICD shock of 0.65 [17]. Our study was inspired by these landmark studies and further demonstrated the potential of ML to reach a higher plateau than traditional statistical modeling. As the amount of clinical data is increasing faster than ever before, it is of vital importance to bring ML into daily practice. ML can exclusively, efficiently, and accurately identify complex patterns from big data. Moreover, it can be easily integrated into electrical medical systems and be updated consistently and automatically.

Our study had several limitations. First, all patients were enrolled in a single center with predominantly secondary prevention indication. Therefore, it may not necessarily be applicable to other populations. Moreover, due to the absence of key variables, we cannot validate and thus make comparisons with established models mentioned before. Nevertheless, as a proof-of-concept study, our primary goal was to illustrate the capacities and advantages of ML and the feasibility of constructing a bi-dimensional risk framework instead of building an out-of-the-box model. Second, appropriate shock may not be a suitable surrogate for life-threatening arrhythmias. Landmark studies have demonstrated only an approximately 50% reduction in SCD in the ICD group compared with placebo [39]. Furthermore, adopting an ICD programming strategy of delayed therapy and high-rate cutoff was associated with reduced inappropriate shocks, unnecessary shocks, and mortality [40,41]. Therefore, appropriate ICD shock may be affected by programming setting and does not necessarily equal life-threatening arrhythmias. However, until now, it has remained the best surrogate endpoint for SCD in clinical research [11,17]. Third, CMR-derived parameters, electrocardiographic measurements, and electrophysiological study results were not available in the study, which showed incremental value in addition to traditional risk factors [22,23,27,32,34,37]. Still, our results showed the capacity of routinely available clinical parameters to derive efficient predictive models. Last, model calibration was not evaluated in the study due to the difficulties of XGBoost and SSVM algorithms in estimating the baseline or cumulative hazard function. This is also a downside of many ML methods implementing survival analysis. In summary, improvements must be made before using these algorithms in clinical settings, including but not limited to increasing sample size, adding more clinically relevant parameters, fine-tuning and optimizing the algorithms, and validating performance in external datasets.

## 5. Conclusions

In this head-to-head comparison of ML and traditional CPH modeling in the risk stratification of ICD recipients, we demonstrated that optimized ML is at least as good as or even better than CPH. A bi-dimensional risk matrix integrating death and shock risk using ML algorithms could facilitate clinical decision-making. This study is exploratory, and further refinement and adjustment are needed before translation into clinical practice.

## Figures and Tables

**Table 1:** Demographics of the training and test sets.

Characteristics		Datasets of All-Cause Death		*p*-Value	Datasets of First Appropriate Shock		*p*-Value
	All Patients (n = 887)	Training Set (n = 665)	Test Set (n = 222)		Training Set (n = 665)	Test Set (n = 222)	
Demographics							
Age (years)	59.0 ± 13.0	59.3 ± 12.8	58.3 ± 13.7	0.361	59.0 ± 13.1	59.1 ± 13.0	0.894
Male sex	667 (75.2%)	504 (75.8%)	163 (73.4%)	0.537	498 (74.9%)	169 (76.1%)	0.779
Body mass index (kg/m^2^)	24.7 ± 3.6	24.8 ± 3.5	24.5 ± 3.8	0.284	24.8 ± 3.7	24.6 ± 3.3	0.631
Ischemic etiology	433 (48.8%)	324 (48.7%)	109 (49.1%)	0.984	317 (47.7%)	116 (52.3%)	0.269
Family history of sudden death	25 (2.8%)	20 (3.0%)	5 (2.3%)	0.723	16 (2.4%)	9 (4.1%)	0.293
Clinical characteristics							
Smoking	416 (46.9%)	316 (47.5%)	100 (45.0%)	0.574	314 (47.2%)	102 (45.9%)	0.802
Primary prevention	240 (27.1%)	185 (27.8%)	55 (24.8%)	0.425	179 (26.9%)	61 (27.5%)	0.94
Dual-chamber ICD	303 (34.2%)	240 (36.1%)	63 (28.4%)	0.044	230 (34.6%)	73 (32.9%)	0.703
Systolic BP (mmHg)	120.5 ± 16.6	120.9 ± 16.4	119.5 ± 17.2	0.298	120.8 ± 16.9	119.7 ± 15.7	0.377
Diastolic BP (mmHg)	73.5 ± 10.3	73.8 ± 10.1	72.9 ± 10.9	0.292	73.5 ± 10.5	73.7 ± 9.8	0.736
NYHA class				0.396			0.498
I	239 (26.9%)	176 (26.5%)	63 (28.4%)		184 (27.7%)	55 (24.8%)	
II	326 (36.8%)	255 (38.3%)	71 (32.0%)		249 (37.4%)	77 (34.7%)	
III	260 (29.3%)	189 (28.4%)	71 (32.0%)		188 (28.3%)	72 (32.4%)	
IV	62 (7.0%)	45 (6.8%)	17 (7.7%)		44 (6.6%)	18 (8.1%)	
Echocardiogram							
LVEDD (mm)	60.3 ± 10.9	60.4 ± 10.8	59.9 ± 11.2	0.606	60.0 ± 11.0	61.2 ± 10.5	0.158
LVEF (%)	43.1 ± 14.6	43.2 ± 14.3	43.0 ± 15.3	0.897	43.4 ± 14.7	42.2 ± 14.1	0.282
LAD (mm)	42.5 ± 8.1	42.5 ± 7.9	42.5 ± 8.8	0.941	42.3 ± 8.0	43.0 ± 8.4	0.306
IVS (mm)	9.4 ± 2.3	9.4 ± 2.2	9.5 ± 2.7	0.555	9.4 ± 2.4	9.4 ± 2.1	0.747
RVD (mm)	22.6 ± 4.3	22.5 ± 4.2	22.9 ± 4.5	0.281	22.7 ± 4.5	22.6 ± 3.7	0.780
Tricuspid valve regurgitation	84 (9.5%)	61 (9.2%)	23 (10.4%)	0.696	67 (10.1%)	17 (7.7%)	0.351
Mitral valve regurgitation	169 (19.1%)	121 (18.2%)	48 (21.6%)	0.305	127 (19.1%)	42 (18.9%)	1.000
Electrocardiogram findings							
Heart rate (beats per minute)	69.0 ± 13.6	68.6 ± 13.7	70.1 ± 13.4	0.163	68.6 ± 13.8	70.1 ± 13.3	0.167
CLBBB	48 (5.4%)	32 (4.8%)	16 (7.2%)	0.232	38 (5.7%)	10 (4.5%)	0.604
CRBBB	53 (6.0%)	42 (6.3%)	11 (5.0%)	0.564	39 (5.9%)	14 (6.3%)	0.939
Frequent PVCs	371 (41.8%)	283 (42.6%)	88 (39.6%)	0.494	281 (42.3%)	90 (40.5%)	0.711
Pacing indication	59 (6.7%)	45 (6.8%)	14 (6.3%)	0.934	44 (6.6%)	15 (6.8%)	1.000
Comorbidities							
Myocardial infarction	345 (38.9%)	266 (40.0%)	79 (35.6%)	0.276	256 (38.5%)	89 (40.1%)	0.732
Atrial fibrillation	259 (29.2%)	190 (28.6%)	69 (31.1%)	0.531	189 (28.4%)	70 (31.5%)	0.425
Hypertension	383 (43.2%)	291 (43.8%)	92 (41.4%)	0.599	285 (42.9%)	98 (44.1%)	0.797
Diabetes	179 (20.2%)	134 (20.2%)	45 (20.3%)	1.000	134 (20.2%)	45 (20.3%)	1.000
Hyperlipidemia	431 (48.6%)	324 (48.7%)	107 (48.2%)	0.954	322 (48.4%)	109 (49.1%)	0.922
Stroke	58 (6.5%)	42 (6.3%)	16 (7.2%)	0.758	48 (7.2%)	10 (4.5%)	0.208
Hyperuricemia	78 (8.8%)	64 (9.6%)	14 (6.3%)	0.169	59 (8.9%)	19 (8.6%)	0.995
Laboratory tests							
NT-proBNP (pg/mL)	788.9 (302.0,1779.0)	765.8 (299.2,1761.8)	853.3 (330.8,1794.2)	0.479	743.6 (299.5,1714.0)	874.8 (316.8,1904.3)	0.268
Hemoglobin (g/L)	140.3 ± 18.1	140.1 ± 17.9	141.0 ± 19.0	0.530	140.8 ± 18.3	138.9 ± 17.8	0.174
Creatinine (μmol/L)	88.0 (75.2,103.7)	87.7 (75.3,104.0)	88.0 (75.0,102.6)	0.955	87.7 (75.0,104.0)	88.3 (75.7,102.9)	0.982
BUN (mmol/L)	6.6 (5.3,8.6)	6.7 (5.4,8.7)	6.0 (4.9,8.3)	0.015	6.6 (5.3,8.6)	6.5 (4.9,8.7)	0.428
hs-CRP (mg/L)	1.9 (0.8,4.6)	2.0 (0.8,4.7)	1.9 (0.8,4.2)	0.962	2.0 (0.8,4.3)	1.7 (0.8,5.6)	0.856
Medications							
ACEI/ARB/ ARNI	573 (64.6%)	426 (64.1%)	147 (66.2%)	0.617	433 (65.1%)	140 (63.1%)	0.637
Amiodarone	461 (52.0%)	354 (53.2%)	107 (48.2%)	0.222	344 (51.7%)	117 (52.7%)	0.862
Beta-blockers	747 (84.2%)	567 (85.3%)	180 (81.1%)	0.170	564 (84.8%)	183 (82.4%)	0.462
Calcium channel blockers	94 (10.6%)	74 (11.1%)	20 (9.0%)	0.446	72 (10.8%)	22 (9.9%)	0.796
Diuretics	564 (63.6%)	428 (64.4%)	136 (61.3%)	0.453	422 (63.5%)	142 (64.0%)	0.956
MRA	524 (59.1%)	396 (59.5%)	128 (57.7%)	0.676	394 (59.2%)	130 (58.6%)	0.919
Digitalis	196 (22.1%)	143 (21.5%)	53 (23.9%)	0.520	150 (22.6%)	46 (20.7%)	0.633
Statin	449 (50.6%)	330 (49.6%)	119 (53.6%)	0.342	332 (49.9%)	117 (52.7%)	0.523
Antiplatelet	322 (36.3%)	248 (37.3%)	74 (33.3%)	0.326	239 (35.9%)	83 (37.4%)	0.758
Anticoagulants	163 (18.4%)	115 (17.3%)	48 (21.6%)	0.180	120 (18.0%)	43 (19.4%)	0.733

Values are presented as the mean ± standard deviation, median (interquartile range), or frequency (%). ACEI/ARB/ARNI, angiotensin-converting enzyme inhibitor/angiotensin receptor blocker/angiotensin receptor-neprilysin inhibitor; BP, blood pressure; BUN, blood urea nitrogen; CLBBB, complete left bundle branch block; CRBBB, complete right bundle branch block; hs-CRP, high-sensitivity C-reactive protein; ICD, implantable cardioverter-defibrillator; IVS, interventricular septum thickness; LAD, left atrial diameter; LVEDD, left ventricular end-diastolic diameter; LVEF, left ventricular ejection fraction; MRA, mineralocorticoid receptor antagonist; NT-proBNP, N-terminal pro-brain natriuretic peptide; NYHA, New York Heart Association; PVC, premature ventricular contractions; RVD, right ventricular diameter.

**Table 2:** Parameter search space and optimal parameters for each model.

Algorithms	Parameter	Search Space	Optimal Parameter for Death Prediction	Optimal Parameter for Shock Prediction
EN-Cox	l1 ratio	0.1, 0.2, 0.3, 0.4, 0.5, 0.6, 0.7, 0.8, 0.9, 1.0	0.9	0.2
	alpha	Log distribution from 0.0001 to 1	0.0233	0.0339
RSF	number of trees	100, 200, 300, 400, 500	400	500
	maximum depth	2, 3, 4, 5, 6, 7	4	7
	minimum samples required to split	10, 14, 28, 22, 40, 50	22	40
	minimum samples required at leaf nodes	5, 7, 9, 11, 20, 25	5	9
SSVM	alpha	0.1, 1, 10, 100	0.1	0.1
	gamma	1, 0.1, 0.01, 0.001	1	0.001
	kernel	rbf, poly, linear, sigmoid, cosine	poly	rbf
	degree (poly kernels only)	2, 3, 4, 5	4	-
XGBoost	loss function	CoxPH	-	-
	learning rate	0.01, 0.05, 0.10	0.1	0.1
	number of trees	20, 25, 30	30	30
	maximum depth	1, 2	2	2
	fraction of samples	0.4, 0.5	0.4	0.4
	fraction of variables	0.4, 0.5	0.5	0.4
	minimum samples required to split	1, 2	1	1

EN-Cox, elastic net Cox regression; RSF, random survival forests; SSVM, survival support-vector machine; XGBoost, eXtreme Gradient Boosting.

## Data Availability

The datasets used and/or analyzed during the current study are not publicly available due to the regulation of Fuwai Hospital.

## Supplementary Materials

The following supporting information can be downloaded at: https://www.mdpi.com/article/10.3390/jcdd9090310/s1, Table S1: Distribution and proportion of missing variables. Table S2: Data before and after Box-Cox transformation and standardization. Table S3: Model performance in the training test. Table S4: Univariable and multivariable Cox regression of all-cause death and first appropriate shock. Table S5: Parameter search space for each model in sensitivity analysis. Figure S1: Continuous variables’ contribution to the outcome in the XGBoost model predicting all-cause death. Figure S2: Continuous variables’ contribution to the outcome in the SSVM model predicting the first appropriate shock. Figure S3: Model interpretability using SHAP values. Figure S4: Comparison of C-index between CPH and ML algorithms in sensitivity analysis. Figure S5: Model interpretability using SHAP values in sensitivity analysis.
