# Supplementary material for: Toward Better Risk Stratification for Implantable Cardioverter-Defibrillator Recipients: Implications of Explainable Machine Learning Models
Source: J Cardiovasc Dev Dis. 2022 Sep 17;9(9):310. doi: 10.3390/jcdd9090310 (PMC9501472; doi:10.3390/jcdd9090310)
Supplement: Supplementary file 1 [file jcdd-09-00310-s001.zip › jcdd-1893489-supplementary.pdf]

Toward better risk stratification for implantable cardioverter-defibrillator recipients: implications of explainable machine learning models

Supplementary material

Authors: Yu Deng*, MD; Sijing Cheng*, MD; Hao Huang*, MD; Xi Liu, MD; Yu Yu, MD; Min Gu, MD; Chi Cai, MD; Xuhua Chen, MD; Hongxia Niu, MD; Wei Hua, MD, Ph.D., FHRS
*These authors contributed equally to this work

Affiliation: The Cardiac Arrhythmia Center, State Key Laboratory of Cardiovascular Disease, Fuwai Hospital, National Center for Cardiovascular Diseases, Chinese Academy of Medical Sciences & Peking Union Medical College, No.167 North Lishi Road, Beijing 100037, China

Correspondence:
Wei Hua, Ph.D.**;** email: huawei@fuwai.com

Table S1. Distribution and proportion of missing variables.
Table S2. Data before and after Box-Cox transformation and standardization.
Table S3. Model performance in the training test.
Table S4. Univariable and multivariable Cox regression of all-cause death and first appropriate shock.
Table S5. Parameter search space for each model in sensitivity analysis.
Figure S1. Continuous variables' contribution to the outcome in the XGBoost model predicting all-cause death.
Figure S2. Continuous variables' contribution to the outcome in the SSVM model predicting the first appropriate shock.
Figure S3. Model interpretability using SHAP values.
Figure S4. Comparison of C-index between CPH and ML algorithms in sensitivity analysis.
Figure S5. Model interpretability using SHAP values in sensitivity analysis.

Table S1. Distribution and proportion of missing variables.

Variables	Number of missing values	Percentage (%)
RVD	34	3.8
hs-CRP	33	3.7
NT-proBNP	21	2.4
IVS	14	1.6
Body mass index	13	1.5
Age	5	0.6
LAD	4	0.5
Hemoglobin	3	0.3
Systolic BP	1	0.1
Diastolic BP	1	0.1
Creatinine	1	0.1
BUN	1	0.1
Heart rate	1	0.1
LVEDD	1	0.1

BP, blood pressure; BUN, blood urea nitrogen; hs-CRP, high-sensitivity C-reactive protein; IVS, interventricular septum thickness; LAD, left atrial diameter; LVEDD, left ventricular end-diastolic diameter; NT-proBNP, N-terminal pro-brain natriuretic peptide; RVD, right ventricular diameter.

Table S2. Data before and after Box-Cox transformation and standardization.

Transformed value<br>Original value	-2	-1	0	1	2
**Dataset for all-cause death**					
Age (years)	30.1	47.3	60.4	71.5	81.4
Body mass index (kg/m2)	18.3	21.3	24.7	28.3	32.2
Systolic BP (mmHg)	91.3	104.6	119.8	137.1	156.8
Diastolic BP (mmHg)	53.5	63.7	73.8	83.9	93.9
NT-proBNP (pg/mL)	30.2	175.5	751.5	2597.0	7653.8
Creatinine (μmol/L)	54.6	67.9	87.5	118.9	175.1
BUN (mmol/L)	3.3	4.6	6.7	10.0	15.4
hs-CRP (mg/L)	0.1	0.5	2.1	6.8	17.4
Hemoglobin (g/L)	103.5	122.3	140.4	157.9	175.0
Heart rate (beats per minute)	46.9	55.8	67.0	81.4	99.9
LAD (mm)	29.0	34.8	41.8	50.2	60.3
IVS (mm)	6.2	7.5	9.1	11.3	14.3
LVEDD (mm)	40.5	49.6	59.8	71.2	83.7
LVEF (%)	18.6	28.9	41.8	57.4	75.9
RVD (mm)	16.4	18.7	21.9	26.3	32.7
**Dataset for first appropriate shock**					
Age (years)	29.2	46.7	60.1	71.6	81.8
Body mass index (kg/m2)	18.2	21.2	24.5	28.4	32.7
Systolic BP (mmHg)	90.0	104.0	119.8	137.6	157.5
Diastolic BP (mmHg)	52.5	63.0	73.5	83.9	94.4

NT-proBNP (pg/mL)	30.1	171.8	740.9	2607.8	7873.5
Creatinine (µmol/L)	54.2	67.7	87.6	118.5	171.3
BUN (mmol/L)	3.2	4.5	6.6	10.0	15.6
hs-CRP (mg/L)	0.1	0.5	2.1	6.6	16.4
Hemoglobin (g/L)	102.6	122.7	141.4	159.0	175.7
Heart rate (beats per minute)	46.6	55.7	67.1	81.5	99.9
LAD (mm)	28.5	34.5	41.7	50.2	60.4
IVS (mm)	6.2	7.4	9.1	11.4	14.7
LVEDD (mm)	40.1	49.0	59.3	70.9	84.1
LVEF (%)	17.6	28.7	42.2	58.2	76.4
RVD (mm)	16.3	18.7	21.9	26.5	33.6

Of note, data preprocessing was independent for the modeling process of all-cause death and appropriate shock. Therefore, transformed values were not necessarily the same in these two datasets.

LVEF, left ventricular ejection fraction; other abbreviations as in Supplemental Table S1.

Table S3. Model performance in the training test

Outcome events	Algorithms				
	CPH	EN-Cox	RSF	SSVM	XGBoost
**Primary analysis**					
**All-cause death**	0.795 (0.755-0.825)	0.784 (0.744-0.819)	0.852 (0.819-0.881)	0.765 (0.728-0.800)	0.831 (0.796-0.867)
**The first appropriate shock**	0.644 (0.607-0.688)	0.652 (0.615-0.693)	0.818 (0.788-0.848)	0.708 (0.673-0.741)	0.715 (0.681-0.754)
**Sensitivity analysis**					
**All-cause death**	0.793 (0.753-0.823)	0.781 (0.741-0.816)	0.874 (0.846-0.898)	0.764 (0.728-0.798)	0.823 (0.791-0.860)
**The first appropriate shock**	0.644 (0.607-0.687)	0.642 (0.606-0.676)	0.842 (0.818-0.865)	0.697 (0.663-0.730)	0.713 (0.678,0.755)

Values are presented as the mean (95% confidence interval).

CPH, Cox proportional hazards; EN-Cox, elastic net Cox regression; RSF, random survival forests; SSVM, survival support vector machine; XGBoost, extreme gradient boosting.

Table S4. Univariable and multivariable Cox regression of all-cause death and first appropriate shock.

	all-cause death				First appropriate shock			
	univariable		multivariable		univariable		multivariable	
Characteristic	Hazard Ratio	p-value	Hazard Ratio	p-value	Hazard Ratio	p-value	Hazard Ratio	p-value
Age	1.36	<0.001	1.24	0.021	0.86	0.033	0.87	0.069
Male sex	1.47	0.064			2.19	<0.001	1.68	0.011
Body mass index	0.94	0.454			0.99	0.879		
Ischemic etiology	1.24	0.201			0.76	0.06		
Family history of sudden death	0.72	0.579			0.62	0.418		
Smoking	1.19	0.29			1.44	0.01		
Primary prevention	1.51	0.018			0.67	0.029	0.53	<0.001
Dual-chamber ICD	0.97	0.883			0.67	0.013	0.73	0.049
Systolic BP	0.81	0.014			0.78	<0.001	0.83	0.021
Diastolic BP	0.85	0.038	0.87	0.088	0.98	0.727		
NYHA class	2.43	<0.001	1.53	<0.001	1.3	0.001		
LVEDD	2.05	<0.001	1.44	0.002	1.44	<0.001	1.23	0.031
LVEF	0.51	<0.001			0.76	<0.001		
LAD	1.96	<0.001	1.22	0.086	1.33	<0.001	1.19	0.044
IVS	0.97	0.702			1.05	0.548		
RVD	1.28	0.004			1.14	0.071		
Tricuspid valve regurgitation	1.59	0.067	0.63	0.1	0.89	0.625		
Mitral valve regurgitation	2.6	<0.001			1.44	0.034		

Heart rate	1.08	0.348			1.11	0.118
CLBBB	1.06	0.879			0.75	0.398
CRBBB	2.39	<0.001	2.16	0.005	0.61	0.237
Frequent PVCs	1.37	0.054			1.06	0.669
Pacing indication	1.61	0.079	1.56	0.111	0.81	0.469
Myocardial infarction	1.31	0.105			0.78	0.109
Atrial fibrillation	2.1	<0.001			1.49	0.007
Hypertension	1.07	0.662			0.8	0.122
Diabetes	2.02	<0.001	1.35	0.119	0.9	0.606
Hyperlipidemia	1.13	0.456			0.89	0.43
Stroke	2.36	0.001			1.45	0.136
Hyperuricemia	1.16	0.584			1.26	0.361
NT-proBNP	2.51	<0.001	1.74	<0.001	1.17	0.04
Hemoglobin	0.89	0.157			1.16	0.035
Creatinine	1.52	<0.001			1.17	0.025
BUN	1.56	<0.001			1.09	0.233
hs-CRP	1.45	<0.001			1.1	0.196
ACEI/ARB/ARNI	1.11	0.528			1.31	0.078
Amiodarone	1.05	0.765			1.46	0.009
Beta-blockers	1.27	0.334			0.98	0.918
Calcium channel blockers	0.83	0.493			0.87	0.561
Diuretics	2.83	<0.001			1.58	0.003
MRA	1.94	<0.001			1.53	0.004
Digitalis	2.24	<0.001			1.26	0.164
Statin	1.07	0.686			0.86	0.28
Antiplatelet	1.08	0.644			0.86	0.312

Anticoagulants	2.05	<0.001	1.39	0.112	1.46	0.029		

Note: all continuous variables were processed with Box-Cox transformation and standardization; one unit change is illustrated in Supplemental Table S2.

ACEI/ARB/ARNI, angiotensin-converting enzyme inhibitor/angiotensin receptor blocker/angiotensin receptor-neprilysin inhibitor; CLBBB, complete left bundle branch block; CRBBB, complete right bundle branch block; ICD, implantable cardioverter-defibrillator; MRA, mineralocorticoid receptor antagonist; NYHA, New York Heart Association; PVC, premature ventricular contractions; other abbreviations as in Supplemental Tables S1 and 2.

Table S5. Parameter search space for each model in sensitivity analysis.

Algorithms	Parameter	Search space	Optimal Parameter for death prediction	Optimal Parameter for shock prediction
EN-Cox	l1 ratio	0.1, 0.2, 0.3, 0.4, 0.5, 0.6, 0.7, 0.8, 0.9, 1.0	0.8	0.1
	alpha	Log distribution from 0.0001 to 1	0.0281	0.1048
RSF	number of trees	100, 200, 300, 400, 500	400	500
	maximum depth	2, 3, 4, 5, 6, 7	5	6
	minimum samples required to split	10, 14, 28, 22, 40, 50	14	22
	minimum samples required at leaf nodes	5, 7, 9, 11, 20, 25	5	7
SSVM	alpha	0.1, 1, 10, 100	0.1	0.1
	gamma	1, 0.1, 0.01, 0.001	1	0.001
	kernel	rbf, poly, linear, sigmoid, cosine	poly	poly
	degree (poly kernels only)	2, 3, 4, 5	4	2
XGboost	loss function	CoxPH	-	-
	learning rate	0.01, 0.05, 0.10	0.1	0.1
	number of trees	20, 25, 30	30	30
	maximum depth	1, 2	2	2
	fraction of samples	0.4, 0.5	0.4	0.4
	fraction of features	0.4, 0.5	0.4	0.4
	minimum samples required to split	1, 2	1	1

Abbreviations as in Supplemental Tables S4.

Figure S1. Continuous variables' contribution to the outcome in the XGBoost model predicting all-cause death.

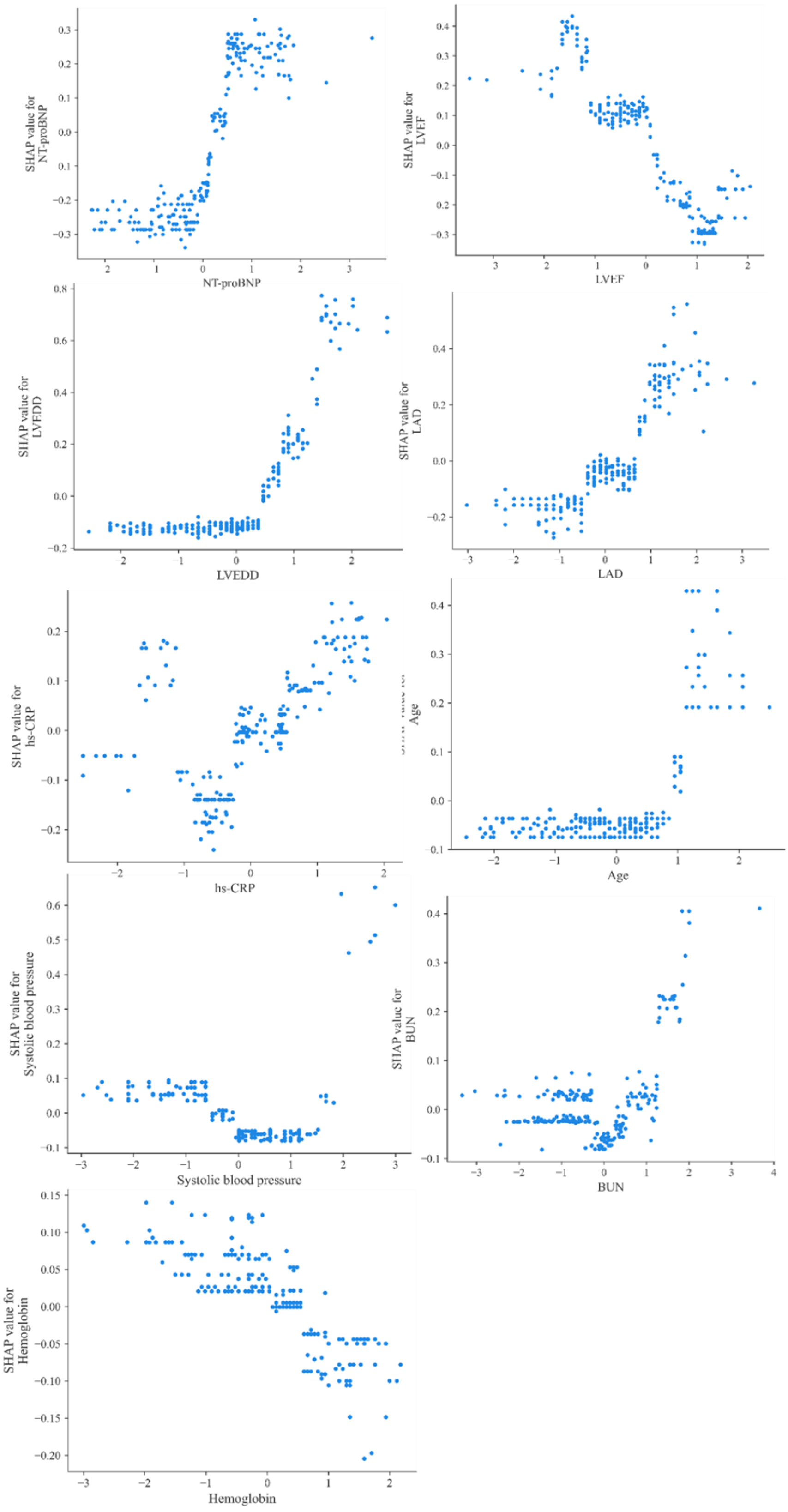

Abbreviations as in Supplemental Tables S1-4.

Figure S2. Continuous variables' contribution to the outcome in the SSVM model predicting the first appropriate shock.

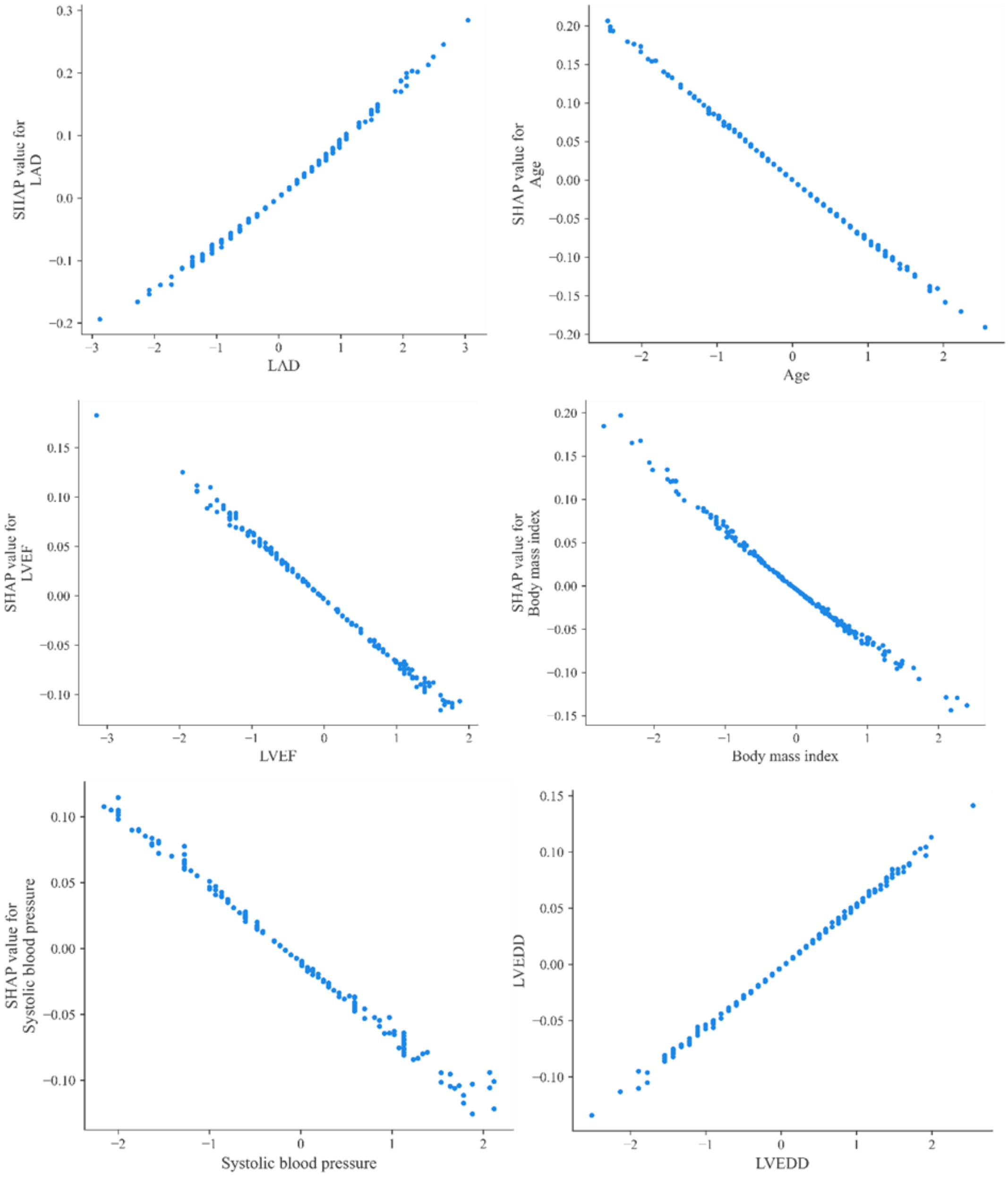

Abbreviations as in Supplemental Tables S1-4.

Figure S3. Model interpretability using SHAP values.

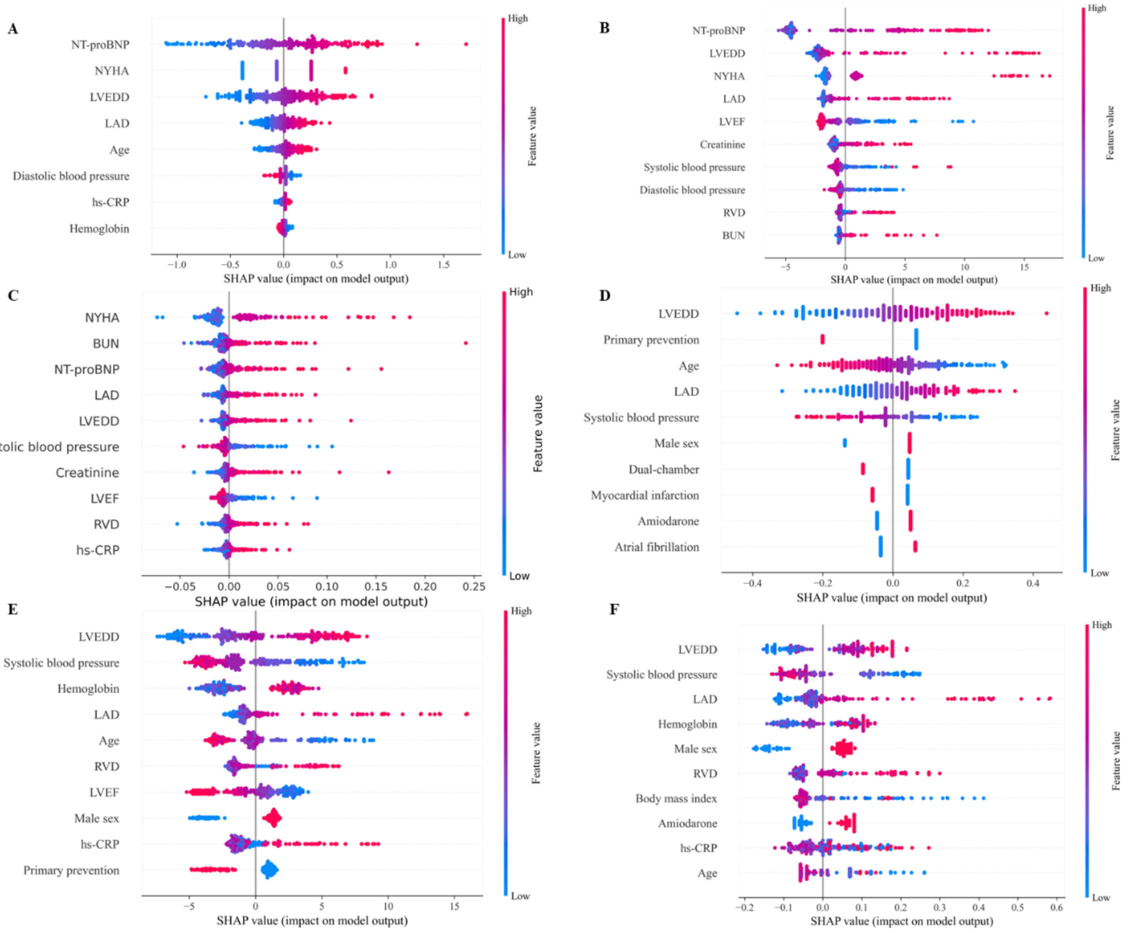

A-C respectively represents SHAP summary plot of EN-Cox, RSF, and SSVM model predicting death; D-F represents SHAP summary plot of EN-Cox, RSF, and XGboost model predicting shock.

SHAP, shapley additive explanation; other abbreviations as in Supplemental Tables S1-4.

Figure S4. Comparison of C-index between CPH and ML algorithms in sensitivity analysis.

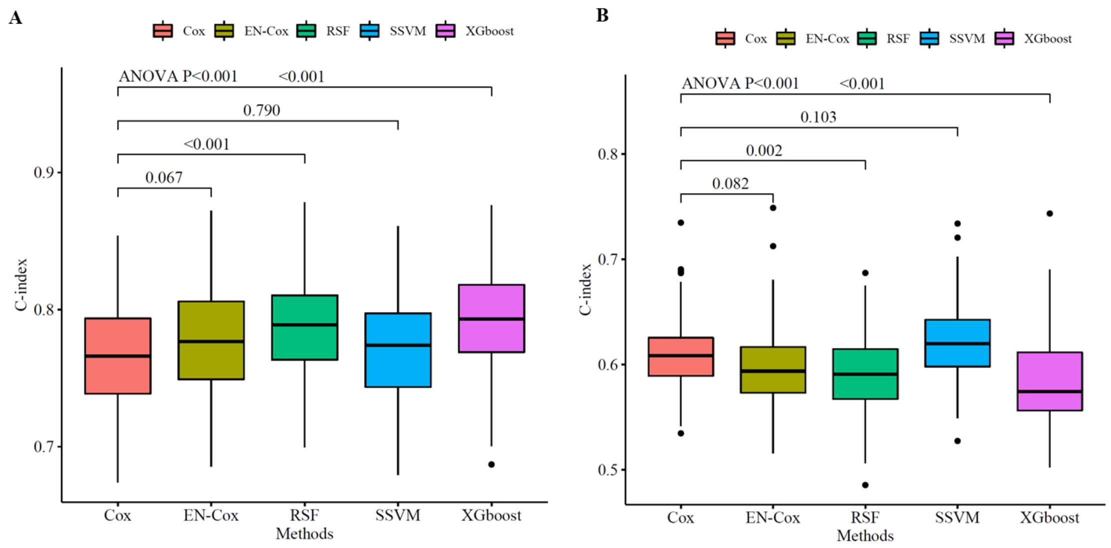

2A and 2B respectively display the comparison of C-index for all-cause death and first appropriate shock in the test set. For predicting all-cause death, the C-index of CPH, EN-Cox, RSF, SSVM, and XGboost were 0.765 (95% CI 0.755-0.773), 0.778 (95% CI 0.770-0.786), 0.787 (95% CI 0.780-0.794), 0.770 (95% CI 0.762-0.778), and 0.790 (95% CI 0.782-0.798), respectively. For predicting shock, the C-index of CPH, EN-Cox, RSF, SSVM, and XGboost were 0.610 (95% CI 0.603-0.617), 0.598 (95% CI 0.590-0.605), 0.591 (95% CI 0.584-0.599), 0.622 (95% CI 0.614-0.629), and 0.584 (95% CI 0.576-0.593), respectively.

CI, confidence interval; other abbreviations as in Supplemental Figure S3 and Table S3.

Figure S5. Model interpretability using SHAP values in sensitivity analysis.

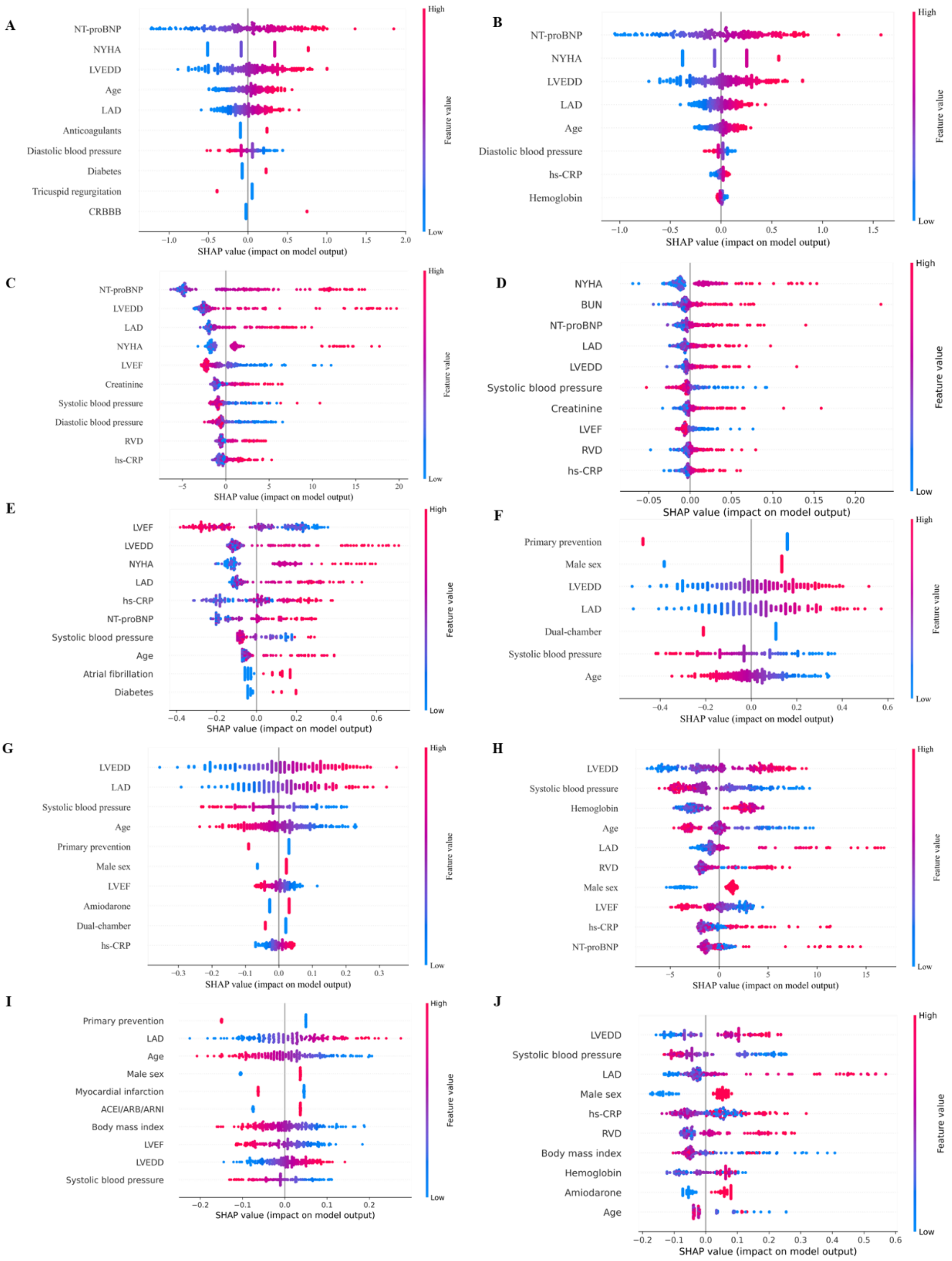

A-E represent SHAP summary plots of CPH, EN-Cox, RSF, SSVM, and XGboost model predicting death; F-J represent SHAP summary plots of CPH, EN-Cox, RSF, SSVM, and XGboost model predicting shock.
Abbreviations as in Supplemental Figure S3, Tables S1-4.
